# Association of LEC and *tnpA Helicobacter pylori *genes with gastric cancer in a Brazilian population

**DOI:** 10.1186/1750-9378-5-1

**Published:** 2010-01-11

**Authors:** Rejane Mattar, Maria S Monteiro, Sergio B Marques, Bruno Zilberstein, Cláudio L Hashimoto, Flair J Carrilho

**Affiliations:** 1 Department of Gastroenterology, University of São Paulo School of Medicine, Av Dr Enéas de Carvalho Aguiar 255, 9° andar sala 9159, São Paulo, 05403-000, SP, Brazil

## Abstract

**Background:**

*H. pylori *seroprevalence in Brazilians varies and is dependent on socioeconomic status, sanitation conditions and ethnicity; furthermore, *H. pylori *is not always associated with the incidence of gastric cancer, suggesting the role of more virulent strains. The purpose of this study was to analyze the association of more virulent *H. pylori *strains with gastric cancer.

**Methods:**

DNA was extracted from gastric biopsies of thirty-four cases of gastric cancer (11 intestinal-type, 23 diffuse-type), and thirty-four of patients with endoscopic gastritis. The presence of *cag*PAI genes (*cagA*, *cagA *promoter, *cagE*, *cagM*, *tnpB*, *tnpA*, *cagT *and the left end of the *cag*II (LEC)) and *babA *were analyzed by PCR.

**Results:**

Comparison of *H. pylori *isolates from gastric cancer and gastritis patients showed significant associations of *tnpA *and LEC with gastric cancer (73.5% [OR, 6.66; 95% CI, 2.30-19.25] and 58.8% [OR, 10.71; 95% CI, 3.07-37.28] of cases, respectively). Other *cag*PAI genes were detected in both groups at similar frequencies.

**Conclusions:**

*tnpA *and LEC of *H. pylori cag*PAI were associated with gastric cancer; nonetheless, these results were restricted within this group of patients and further studies are needed to confirm these results in a larger sample and determine their role in gastric carcinogenesis.

## Findings

The seroprevalence of *H. pylori *ranges among Brazilians, and is dependent on age, socioeconomic status, and ethnicity, as well as sanitation conditions. High prevalence has been reported in the city of Fortaleza, with 73.3% positive cases in individuals 11-20 years old, and up to 87% in those over 60 years old [1]. Notably, the incidence rate of gastric cancer is lower (16/100,000) in Fortaleza than in the city of São Paulo (22/100,000) http://www.inca.gov.br/, which has a lower prevalence of *H. pylori *(65.6%) [2]. Furthermore, *H. pylori *seroprevalence was higher in African Brazilians compared to Caucasoids [2] and Japanese Brazilians [3], despite the higher mortality for gastric cancer among Japanese Brazilians compared to the indigenous population [4]. These conflicting results suggest among other factors that more virulent *H. pylori *strains may be involved in gastric cancer outcome.

The *cag *(cytotoxin-associated gene) pathogenicity island (*cag*PAI) in *H. pylori *contains 31 putative genes [5] and encodes a type IV secretion system that delivers CagA into the cytosol, which is phosphorylated and activates phosphatase activity to initiate morphological changes of the cell, providing a potential mechanism by which chronic *H. pylori *infection may promote the development of gastric cancer [6]. We previously showed that the *cag*PAI genes *cagT*, *cagM*, *cagA *and LEC (the left end of the *cag*II) were significantly associated with peptic ulcers [7], and these findings were later confirmed by another group in Brazil [8]. The purpose of this study was to analyze the association of *cag*PAI genes and the blood group antigen binding adhesin *babA *of *H. pylori *with gastric cancer.

Patients were from the indigenous population of the city of São Paulo and were classified according to ethnicity as White, Brown (of White and African-Brazilian descent) and Black, none was Japanese-Brazilian. Among 68 consecutive gastric cancer patients, only 34 (11 intestinal-type, 23 diffuse-type) were *H. pylori *positive, 20 of which were men and 14 were women; the patients' mean age was 53.3 ± 12.4 years, and ranged from 24 to 75 years old; the median age was 52.5 years; 25 were White, 7 were Brown and 2 were Black.

Controls were selected among dyspeptic patients without previous history of peptic ulcer and gastric cancer, presenting a recent diagnosis of only gastritis (superficial or erosive) by upper gastrointestinal endoscopy, and positive for *H. pylori *infection. Of the 34 controls, 23 were women and 11 were men, with a mean age of 50.9 ± 9.8 years and a range from 32 to 70 years old; the median age was 48.5 years; 29 were White, 4 were Brown and 1 was Black.

The criteria for *H. pylori *positivity were the same for patients and controls, positive urease test and PCR with primers that amplify the species-specific 26-kDa antigen gene, using antrum and corpus biopsies [7,9]. Patients and controls had not taken antimicrobials and acid suppression drugs for at least 30 days before the sample collection. In patients with gastric cancer, fragments were obtained from the normal appearance mucosa either by upper gastrointestinal endoscopy or after gastrectomy, immediately after opening the stomach. All patients provided informed written consent, and this study was approved by the local Ethics Committee.

DNA extraction from gastric biopsies of the positive urease tests and PCR were performed according to previously reported techniques. PCR analysis amplified regions of the *cag*PAI genes, *babA*, and DNA sequence of a 26-kDa species-specific protein antigen present in all strains of *H. pylori *[7,9]. All the cases were confirmed *H. pylori *positive by urease test and by PCR for the species-specific antigen.

Fisher's exact probability test was determined using SPSS, and the odds ratio [OR] and 95% confidence interval [95% CI] were calculated using Microsoft Office Excel 2003. A value of *P *< 0.05 was considered statistically significant. The [OR] and [95% CI] are depicted in Table 1.

**Table 1:** Prevalence of *cag*PAI genes and *babA *in *H. pylori *isolates from gastritis and gastric cancer patients.

Genes	Controls (n = 34)	Gastric cancer (n = 34)	*P *value	OR	95% CI
***cagA***	8 (23.5%)	12 (35.3%)	*P *= 0.287	1.77	0.61-5.11
***cagE***	24 (70.6%)	22 (64.7%)	*P *= 0.604	0.76	0.27-2.11
***cagM***	8 (23.5%)	6 (17.6%)	*P *= 0.549	0.69	0.21-2.27
***cagT***	16 (47.1%)	16 (47.1%)	*P *= 1.000	1.00	0.38-2.59
**ap*cag***	20 (58.8%)	23 (67.6%)	*P *= 0.451	1.46	0.54-3.94
**LEC**	4 (11.8%)	20 (58.8%)	*P *< 0.0001	10.71	3.07-37.28
***tnpA***	10 (29.4%)	25 (73.5%)	*P *< 0.0001	6.66	2.30-19.25
***tnpB***	1 (2.9%)	2 (5.9%)	*P *= 0.551	2.06	0.17-23.88
***babA***	22 (64.7%)	14 (41.2%)	*P *= 0.052	0.38	0.14-1.01

*P *< 0.0001 by Fisher's exact probability test

Comparison of the presence of *cag*PAI genes in *H. pylori *isolates from patients with gastric cancer and gastritis revealed that only two genes were significantly associated with gastric cancer: *tnpA*, detected in 73.5% (25/34) of the gastric cancer cases, with an odds ratio of 6.66 [95% CI, 2.30-19.25], and LEC, observed in 58.8% (20/34) of cases, with an odds ratio of 10.71 [95% CI, 3.07-37.28]. The other *cag*PAI genes analyzed in this study were detected equally in both groups. *cagA *was more frequently found in the gastric cancer group, though this difference was not significant; however, its presence may still be associated with gastric cancer, due to the odds ratio of 1.77 [CI 95%: 0.61-5.11]. The *babA *gene was more often found in *H. pylori *isolates from patients with gastritis (64.7%, 22/34) than in isolates from gastric cancer (41.2%, 14/34; p = 0.052), [OR, 0.38; 95% CI, 0.14-1.01]. The analysis of *cag*PAI and *babA *genes among the histological types (diffuse and intestinal) of gastric cancer, gender and the ethnic groups revealed no significant difference, data not shown.

As shown in Table 2, *H. pylori *isolates from gastric cancer cases were usually positive for both LEC and *tnpA *(*p *= 0.017 by Fisher's exact test). In Japan association of IS*605 *with other *cag*PAI genes, *cag13 *and *cagA *was previously reported in gastric cancer cases. However, as the role of this finding remains unclear, further study is necessary to determine its involvement in gastric cancer [10].

**Table 2:** Association of *tnpA *and LEC in *H. pylori *isolates from patients with gastric cancer.

Gastric cancer patients (n = 34)	*tnpA *-	*tnpA *+	Total
LEC -	**7**	**7**	14
LEC +*	2	18*	20

**Total**	**9**	**25**	**34**

*p = 0.017 by Fisher's exact probability test

In this study, we analyzed the prevalence of *H. pylori *genes in patients from a Brazilian population with gastric cancer or gastritis. Unexpectedly, *babA*, the blood-group antigen binding adhesion targeting human Lewis^b ^surface epitopes on gastric epithelial cells associated with duodenal ulcer and gastric adenocarcinoma [11], was frequently deleted in gastric cancer *H. pylori *isolates (41.2%), compared to gastritis (64.7%); nonetheless, in the previously studied peptic ulcer group, 69.3% of *H. pylori *isolates also were *babA *positive [9]. Thus, *babA *may be a frequent genotype with no particular role in the clinical outcome [12].

We previously showed that the *cag*PAI genes *cagT*, cag*A*, *cagM *and LEC were associated with peptic ulceration progression [7]; this study revealed that only LEC and *tnpA *were associated with gastric cancer. In South Africa, LEC was frequently deleted in isolates from gastritis compared to those from gastric cancer and peptic ulcers [13]. Nevertheless, LEC was not necessary for either the translocation of CagA that mediates intracellular disruption of growth regulation [14], or for the induction of IL-8 [15], proinflammatory cytokine released upon *H. pylori *infection by gastric epithelial cells that induced expression and activation of epidermal growth factor receptor and proliferation [16]. Our finding is consistent with a previous study reporting higher frequency of *tnpA *in Peruvian gastric cancer strains than in gastritis strains (9 of 14 versus 15 of 45, respectively; *P *= 0.04) [17].

We did not observe any significant association of *cagA *with cases of gastric cancer or gastritis, and this is consistent with previous reports in South Africa [13] and Germany [18], which failed to detect an association with gastric cancer.

Our study of a population in Brazil indicates an association with LEC and *tnpA *and gastric cancer; nonetheless, further studies are needed to confirm these results in a larger sample, as no specific role for these genes in gastric carcinogenesis has yet been identified.

## Abbreviations

*cag*PAI: cytotoxin-associated gene pathogenicity island; LEC: left end of *cag*II region

## Competing interests

The authors declare that they have no competing interests.

## Authors' contributions

RM participated in the conception, design, analysis and interpretation of data, statistical analysis, drafted the manuscript and revised final version; MSM carried out the molecular genetic studies; SBM and CLH obtained gastric biopsies; BZ provided gastric cancer patients and presented the study at the 8^th ^International Gastric Cancer Congress; FJC gave final approval of the version to be published. All authors read and approved the final manuscript.
